# Magnetic 3D-Printed Composites—Production and Applications

**DOI:** 10.3390/polym14183895

**Published:** 2022-09-17

**Authors:** Guido Ehrmann, Tomasz Blachowicz, Andrea Ehrmann

**Affiliations:** 1 Virtual Institute of Applied Research on Advanced Materials (VIARAM);; 2 Institute of Physics—Center for Science and Education, Silesian University of Technology, 44-100 Gliwice, Poland; 3 Faculty of Engineering and Mathematics, Bielefeld University of Applied Sciences, Interaktion 1, 33619 Bielefeld, Germany

**Keywords:** fused deposition modeling (FDM), magnetic hydrogel, photopolymerization, stereolithography (SLA), hydrogel, shape memory polymer (SMP), magneto-rheological behavior, electromagnetic shielding

## Abstract

Three-dimensional printing enables building objects shaped with a large degree of freedom. Additional functionalities can be included by modifying the printing material, e.g., by embedding nanoparticles in the molten polymer feedstock, the resin, or the solution used for printing, respectively. Such composite materials may be stronger or more flexible, conductive, magnetic, etc. Here, we give an overview of magnetic composites, 3D-printed by different techniques, and their potential applications. The production of the feedstock is described as well as the influence of printing parameters on the magnetic and mechanical properties of such polymer/magnetic composites.

## 1. Introduction

Three-dimensional printing belongs to the most important new technologies of our time, enabling building objects with special shapes and properties in a fast and often low-cost manner [1,2]. Three-dimensional printing techniques are being developed further to improve accuracy and mechanical properties, reduce surface roughness, and enable producing even more shapes in a shorter time [3,4]. Recently, resin and photopolymer-based techniques are especially gaining more interest as they enable the creation of very small structures in a reliable way [5,6,7]. Nevertheless, fused deposition modeling (FDM) and similar techniques working with molten polymer often have the advantage of lower toxicity and reduced post-processing, as compared to typical stereolithography (SLA) and similar processes, and they are, thus, often favored for use at home or at school or university [8,9,10].

**Figure 1:**
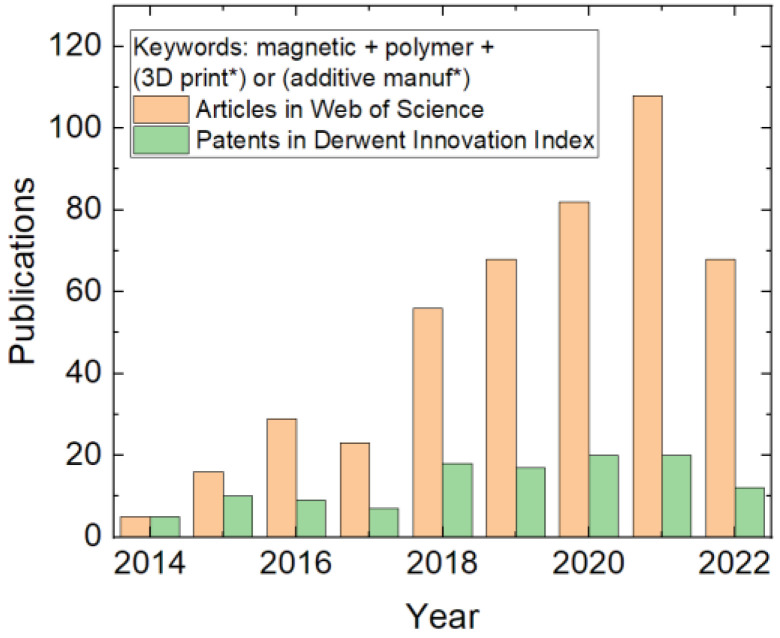
Search results in the Web of Science and the Derwent Innovations Index for the named keyword combinations, counted on 24 August 2022.

Another possibility to improve 3D-printed objects’ functionality and, thus, to increase the possible areas of application is based on modifying the materials, ideally without the necessity to change the printing process itself. This can be performed by adding a second phase to the polymer feedstock, e.g., in the form of nanofibers or nanoparticles, in this way creating a composite material [11,12,13]. Such a composite feedstock is often prepared to improve the mechanical properties of a 3D-printed object [14,15]. However, it is also possible to make 3D-printed objects conductive, at least to a certain amount [16,17,18], or make them magnetic by adding suitable nanofibers or nanoparticles. While magnetic 3D-printed composites are not as often investigated as conductive ones, an increasing trend is visible in the scientific literature and patents (Figure 1), showing the growing interest of researchers in materials with magnetic properties that is based on the broad range of technical, biomedical, and other applications, as described in Section 5.

This review gives an overview of magnetic 3D-printed composites. The paper is structured as follows: After a short summary of different 3D printing techniques, the preparation of a magnetic feedstock for FDM, SLA, and other technologies is discussed. Technical applications as permanent magnets of special shapes are discussed, as well as medical and other applications. Results of the most recent basic research in these composites as well as corresponding simulations are presented. Finally, going from the material aspect further to modified printing technologies, the magnetic-field-supported 3D printing is described as a special 3D printing technique for extrusion- and photopolymer-based technologies. To avoid neglecting the most recent (i.e., not yet Web of Science indexed) papers, the keywords in Figure 1 were used for a search in Google Scholar. Papers with unclear experimental information or very similar contents to already described materials, processes, or applications were not taken into account, to avoid unnecessary repetitions without further information for the reader.

## 2. Three-Dimensional Printing Techniques

This section gives a compact overview of the most important 3D printing techniques. Generally, the feedstock for 3D printing can take the form of a filament/wire (e.g., FDM printing), a powder (e.g., selective laser sintering, SLS), or a fluid (e.g., SLA, PolyJet Matrix [19]), in addition to two- or more-component methods in which a binder and a powder or other components are combined (e.g., binder jetting [20]). Depending on the exact way of processing the feedstock, several technologies can be defined.

FDM is most common in inexpensive printers used at home or in school or university. A polymer filament of defined diameter is pulled into a hot-end, molten, and pressed through a nozzle. The molten polymer is placed along defined paths to form an object layer by layer [2,9]. For metals, a similar process is wire arc additive manufacturing [21].

SLS works by fusing subsequent layers in a powder bed and can not only be used to process polymers, but also metals or ceramics [22,23]. Other powder-bed-based techniques are, e.g., selective laser melting, direct metal laser sintering, and electron beam melting [24].

Among the photopolymerization techniques, SLA is the oldest and probably the most common one. In this technique, the printing bed is lowered into a basin filled with resin, and the highest layer is polymerized by laser light, before the printing bed is lifted by one layer height, and the next layer is polymerized [25]. Digital light processing (DLP) uses a digital micro-mirror device in the light path, enabling light exposure of a full layer at the same time [26]. In the PJM technology, objects are created on top of a printing bed, where a liquid polymer resin is sprayed on desired positions and cured by UV light [19]. Even more exact shapes can be reached by two-photon or multi-photon polymerization, where a strongly focused laser enables absorbing two or more photons simultaneously, in this way strictly limiting the area where polymerization can take place [27,28].

In addition to the aforementioned techniques, several others exist, some of which can be related to printing polymers. Such more special technologies are explained in brief in the following sections, where necessary.

## 3. Magnetic Feedstock for Fused Deposition Modeling (FDM)

Generally, many magnetic materials can be embedded in a polymeric feedstock for FDM printing, from nanoparticles to microflakes or fibers in different dimensions, and from soft to hard magnetic materials. Similarly, diverse polymers can be applied, most of them typical for FDM printing. Common soft magnetic materials used, e.g., in electric machines to guide magnetic fields, are Si-Fe alloys (also printable without a polymer matrix [29]), Ni-Fe alloys (e.g., permalloy [30]), nickel [31], magnetite [32,33], NiZn ferrites [34,35], MnZn ferrites [36], etc. Hard magnetic materials, characterized by large coercivity and ideally also large remanence, are used for technical permanent magnets and often include rare-earth elements, such as neodymium-iron-boron (NdFeB)-based magnets [37] or magnetic ceramics such as barium hexaferrite [38] or strontium hexaferrite, while manganese-based alloys such as MnBi, MnAl, or MnGa, alnico magnets, and iron nitride (Fe_16_N_2_) are less often used in 3D-printed polymer/magnetic composites.

**Figure 2:**
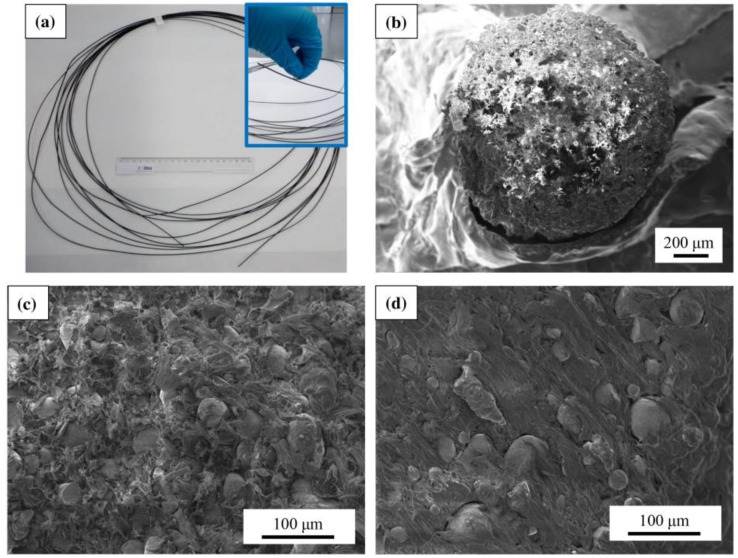
(**a**) Extruded MnAlC–PE magnetic filament (with a 20 cm ruler for scale comparison); SEM images of (**b**) the circular cross-section of the MnAlC-PE filament, and internal filament morphology for filling factors (**c**) 72.3% and (**d**) 52.1%. From [40], originally published under a CC-BY license.

Due to the interest in 3D printing freeform magnets with different magnetic properties, several research groups developed a magnetic feedstock for FDM printing, often based on polymer/ferrite composites [39]. Palmero et al. described the preparation of a polyethylene (PE)/MnAlC composite filament [40]. For this, they prepared a magnetic MnAlC powder by gas-atomization, resulting in spherical particles of diameters below 36 µm, which were embedded in a PE matrix by dispersing/dissolving both materials in toluene, so that a polymeric matrix was formed around the MnAlC particles. The resulting pellets were extruded into a magnetic filament with a diameter of 1.75 mm, suitable for standard FDM printing, and showed coercive fields around 1.5 kOe [40]. The filament and its inner structure are depicted in Figure 2. The research group showed in a subsequent paper a filling factor of even 80 wt%, which they attributed to an optimum ratio of fine to coarse particles, enabling the production of freeform permanent magnets without rare earth materials [41].

The production of a magnetic FDM feedstock containing polyethylene was also reported by Arbaoui et al. who used permalloy, or more exactly, Ni_81_Fe_19_, as magnetic particles [42]. These commercially available spherical particles had diameters of a few microns, i.e., smaller than those used by Palmero et al. [40,41]. Composite materials were prepared by propeller mixing in a molten state as well as by using a twin screw extruder, with filler volume fractions between 10% and 40%. From the composite with 30 vol% permalloy, an FDM filament with a diameter of 3 mm was prepared and used to print a small parallelepiped, which was not further investigated [42].

**Figure 3:**
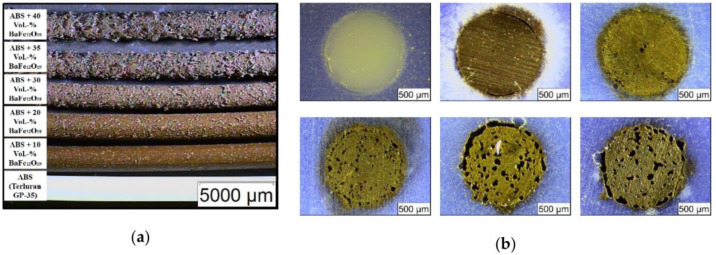
Microscopic images of (**a**) extruded filaments with different solid loadings; (**b**) polished cross-sections with increasing filler content. Upper row: Pure acryl butadiene styrene (ABS), 10 vol.% and 20 vol.% ferrite; lower row: 30 vol.%, 35 vol.%, and 40 vol.% ferrite. From [43], originally published under a CC-BY license.

Another polymer often used to prepare magnetic FDM feedstock is acryl butadiene styrene (ABS). Hanemann et al., e.g., prepared a composite feedstock from ABS and barium ferrite (BaFe_12_O_19_), which is a ferrimagnetic material [43]. The commercially available barium ferrite had an average particle size of 500 nm, i.e., much smaller than the previously discussed MnAlC or permalloy microparticles. The composite material was produced in a mixer-kneader unit by adding a surfactant to improve the surface coverage of the nanoparticles, before a filament extruder was used to prepare the final FDM filament with a diameter of 1.75 mm. Among these filaments (Figure 3), those with up to 30 vol% barium ferrite could be used to print samples with sufficient quality on a commercial FDM printer. Coercivities were found in the range of 11 kA/m for 10 vol% barium ferrite up to 29 kA/m for 35 vol% barium ferrite.

Gas-atomized stainless-steel alloy particles of diameters around 25 µm were also mixed with ABS to prepare a composite feedstock [44]. For this, ABS pellets were dissolved in acetone, before the metallic particles were added under stirring, and stirring was continued until the acetone was fully evaporated. The resulting composites had stainless-steel volume ratios of 50–90% and were further extruded to filaments with a diameter of 1.75 mm. The filaments showed small coercive fields around 52–55 Oe, with the values of the printed objects being very similar to those of the original stainless-steel powder. While FDM printing from these filaments was successful, the authors nevertheless mentioned future approaches to improve the resolution of the 3D-printed objects.

A similar study with stainless-steel micropowder with an average diameter of 6 µm, compounded in ABS, was performed by heated kneading of ABS and the steel micropowder, followed by extrusion of FDM filaments with a diameter of 1.75 mm [45]. The authors report printing with these filaments for magnetic fillers up to 40 vol%. Coercivities were found around 1.5–4.3 kA/m in this investigation.

Other combinations of polymers and magnetic nano- or microparticles are also reported in the literature. Amongst them, poly(lactic acid) (PLA) was blended with magnetite (Fe_3_O_4_) and partly with additional poly(vinyl chloride) (PVC) and wood powder [46,47,48]. From the feedstock prepared by twin screw extrusion, filaments with 20 wt% magnetite were prepared and investigated in terms of mechanical and magnetic properties. They showed coercive fields around 84 Oe for PLA as the matrix and of approx. 75 Oe for the hybrid matrix.

While the aforementioned coercive fields are relatively small, much larger values can be found for rare-earth magnetic alloys, such as NdFeB. Pigliaru et al. mixed the high-temperature polymer poly(ether ether ketone) (PEEK) with NdFeB flakes of average thickness 35 µm and coercive fields of 440–496 kA/m after magnetization [49]. Filaments with a diameter of 1.75 mm were prepared by mixing both components in a planetary mixer, followed by single-screw extrusion at 340 °C. The filaments contained 25–75 wt% of NdFeB. For FDM printing, a special high-temperature printer from Indmatec was used to reach the necessary nozzle temperature of 100 °C. The authors reported that while 25 wt% of NdFeB was still printable with the common nozzle diameter of 0.4 mm, this had to be changed to a nozzle with a diameter of 0.8 mm for the filament containing 50 wt% NdFeB due to the high viscosity of this melt, and the filament with 75 wt% NdFeB could not be printed successfully. Interestingly, while the printed samples with 25 wt% NdFeB had a similar coercive field as the original powder of 413 kA/m, the sample with 50 wt% NdFeB had a significantly reduced coercive field of approx. 167 kA/m, which the authors attributed to a reduced orientation of the magnetic flakes, in this case, due to the larger nozzle.

As these examples show, different combinations of polymer and magnetic nano-/microparticles were shown to be suitable as a feedstock for magnetic FDM filaments. However, while FDM printing has a broad area of applications, the finer structures available by photopolymerization may be more suitable for well-defined magnetic object shapes. The next section, thus, introduces possibilities to prepare magnetic photopolymers, but also other feedstock for different 3D printing techniques.

## 4. Other Magnetic Feedstock

While the preparation of a magnetic FDM filament necessitates a filament extruder of sufficient quality, adding magnetic nanoparticles to a photo-curable resin seems to be more straightforward. Some research groups report such approaches to prepare magnetic resins for SLA and similar 3D printing techniques.

**Figure 4:**
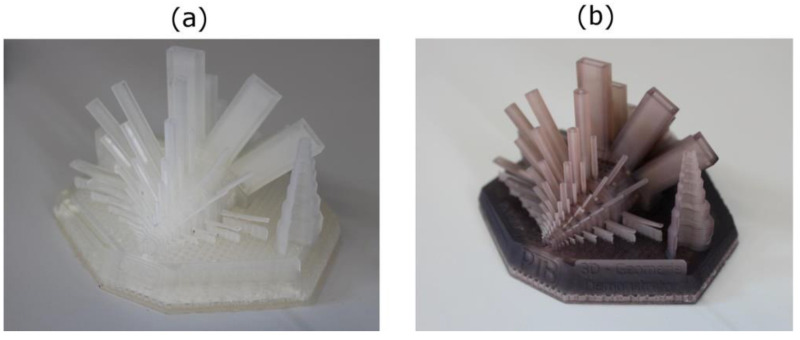
Three-dimensionally printed demonstrator using E-Shell without MNP (**a**) and with MNP (**b**) at a concentration *c*(of Fe) = 3.5 mg/mL. From [50], originally published under a CC-BY-NC-ND license.

Löwa et al. discussed the addition of magnetic iron oxide nanoparticles and a ferrofluid to two different photopolymers, namely E-shell 600 clear and ABS 3SP Tough [50]. They report that sonication of the photopolymer while the nanoparticles were added supported stability and homogeneity of the mixture. In addition, the final blend was degassed in an ultrasonic bath. These mixtures were printed in a commercial DLP printer. The authors mention an estimated nanoparticle change of less than 10% during some hours of printing, based on settling of the nanoparticles, especially for mixtures prepared without sonication. Figure 4 depicts the E-shell photopolymer without and with magnetic nanoparticles, indicating that, in some cases, the printing of thin rods could even be improved by addition of magnetic nanoparticles. Magnetic particle imaging showed a slightly better resolution for the nanoparticles printed from ABS photopolymer, as compared to E-shell.

Instead of nanoparticles, Ren et al. inserted magnetic microfibers into a clear photosensitive resin [51]. For this, micrometer-sized nickel-coated carbon fibers were modified with a silane coupling agent and mixed with the resin as well as a small amount of fumed silica as a tackifying agent. Stirring was performed mechanically, followed by degassing by applying alternately ultrasonication and vacuum (10 mbar). By applying an external magnetic field during DLP printing with varying orientation from layer to layer, the authors reported improved mechanical properties for sophisticated fiber orientation distributions. This idea of printing inside a magnetic field is discussed more in detail in Section 7.

**Figure 5:**
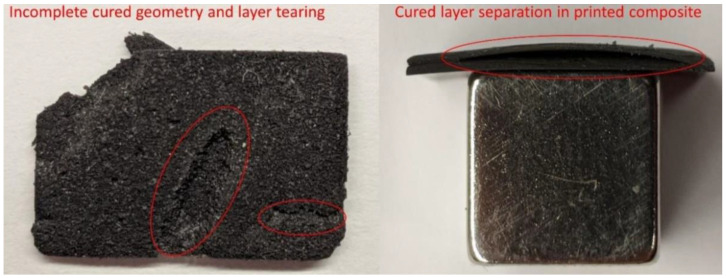
Defects in SrFeO (**left**) and NdFeB composite (**right**) printed with resins containing 25 wt% magnetic fillers. Reprinted from [52], copyright (2020), with permission from Elsevier.

Instead of these common magnetic elements, Nagarajan et al. used strontium ferrite (SrFe_12_O_19_) powder with an average diameter of 1.41 µm and neodymium iron boron (Nd_2_Fe_14_B) powder with an average diameter of 5 µm as ferromagnetic additions to a UV-curable urethane acrylate resin [52]. Mixing was performed mechanically and by sonication, while two different additives were applied to stabilize the dispersion with different amounts of magnetic filler. During DLP printing, different UV projections and, thus, exposure energies as well as other parameters were tested. They found that the thickness of the printed thin samples depended especially on the loading with magnetic particles, the layer thickness, and the waiting time before exposure. Samples with 10 wt% of NdFeB or SrFeO could be printed well, while 25 wt% magnetic particles resulted in partly separated layers, as depicted in Figure 5. In dimensional tests, all printed samples were slightly broader than the target width, and the minimum width that could be printed was found to be 0.13 mm.

Another possibility to prepare a magnetic feedstock for a 3D printing process is preparing a powder blend for powder bed fusion or similar powder-based 3D printing processes. Instead of mixing polymer and metal powder, Hupfeld et al. described a process to coat polyamide powder with superparamagnetic FeO_x_ nanoparticles that they prepared by laser fragmentation in a liquid environment [53]. The magnetic properties of the nanoparticles were not influenced by the powder bed fusion process, making this process suitable for the preparation of 3D-printed objects by powder-based processes.

Magnetic hydrogels are also highly interesting for diverse applications, from medical purposes to soft robotics and actuators in general [54]. One possibility to create magnetic hydrogels is by adding Fe_3_O_4_ nanoparticles in aqueous solution to sodium alginate, followed by adding CaCl_2_ to prepare the hydrogel precursor, adding methylcellulose at 80 °C, and cooling down the blend [55]. In this way, Podstawczyk et al. created a thixotropic ink that kept its shape directly after printing by a bioprinter. Similarly, Li et al. introduced magnetic graphene oxide nanoparticles, including Fe_3_O_4_, into an alginate hydrogel containing poly(vinyl alcohol) (PVA) and hydroxyapatite for the possible use as scaffolds for bone regeneration [56].

Finally, it should be mentioned that magnetic 3D-printed objects can also be produced in a two-step procedure by first printing, followed by coating with a magnetic material, e.g., by electroless coating [57]. However, most reports about magnetic 3D-printed objects deal with fully magnetic samples, prepared by one of the aforementioned techniques.

## 5. Applications

**Figure 6:**
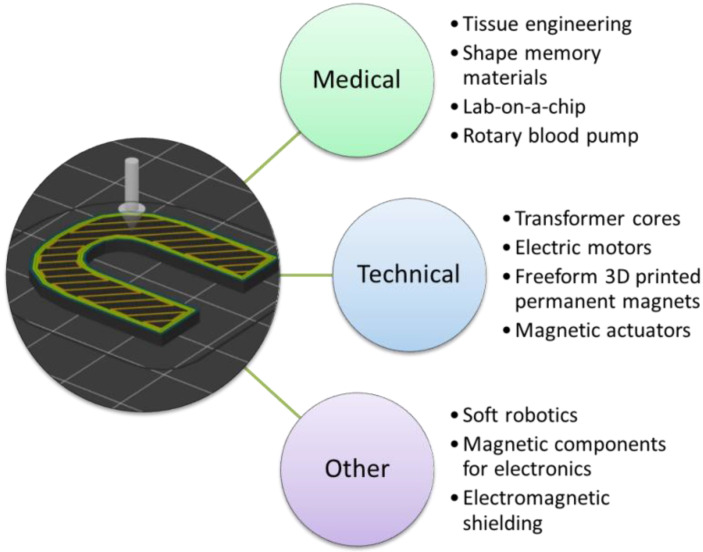
Overview of potential fields of application for 3D printed magnets.

The aforementioned techniques to use magnetic material in 3D printing can be used for diverse applications, from transformer cores with special shapes to magnetic scaffolds for tissue engineering. A broad overview of possible fields of applications is given in Figure 6, before a more detailed description of special projects is given in the following subsections.

### 5.1. Technical Permanent Magnets

**Figure 7:**
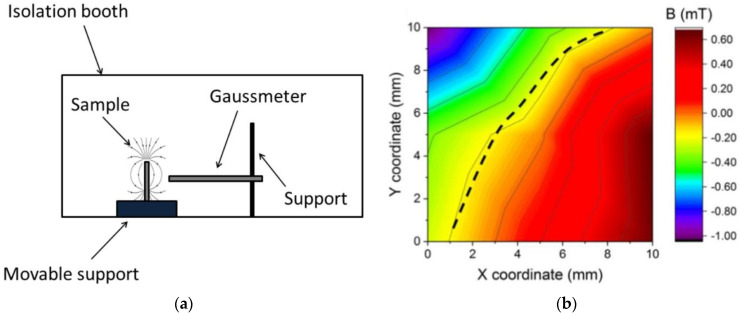
(**a**) Experimental setup used to measure the magnetic field generated by 3D-printed samples, located inside a magnetically isolated booth to minimize magnetic noise; (**b**) magnetic field orientation and intensity after magnetic poling of 3D-printed big square thin. Reprinted from [58], copyright (2018), with permission from Elsevier.

To enable technical usability of 3D-printed magnets, it is necessary to characterize the printed objects in detail. This is especially important for magnets with unusual shapes, where calculation of the complete magnetic field from a single measurement is not as easy as for highly geometric shapes. For this, Domingo-Roca et al. suggested the setup depicted in Figure 7a and the received maps such as the one shown in Figure 7b, here for an SLA-printed thin large square [58]. The dashed line in Figure 7b corresponds to a change in magnetic polarity according to the poling of the sample.

According to the planned application, diverse materials can be found in 3D-printed magnetic composites for technical magnets. NdFeB particles were, e.g., embedded in a thermoset epoxy-based ink that was processed by direct-write 3D printing [59]. Compton et al. reported printing ring, bar, and horseshoe-shaped 3D-printed magnets that were cured at 100 °C without losing their magnetic properties. As mentioned before for another group [52], Palmero et al. also used NdFeB as well as strontium ferrite to produce 3D-printed magnets by extrusion of the magnetic/polymer composite materials [60]. They reported extrusion through nozzles with different shapes, suggesting the extruded strips with defined cross-sections as final products or as filaments for FDM printing.

Bollig et al. described possibilities to 3D-print transformer cores with ideal geometries [61]. They used the commercially available “PLA Rustable Magnetic Iron” filament from Proto-Pasta with 40 wt% iron, which they printed in a common FDM printer with a special nickel-plated brass nozzle to avoid abrasion due to the iron content in the filament. Cores in toroidal shape were printed with different fill patterns and fill factors, before wires were wrapped around them to enable comparison with commercial cores in a test circuit. The authors found coercive fields of the filament of <10 Oe, i.e., a very soft magnetic material, as necessary for transformer cores. Nevertheless, the printed core could not be saturated in the same way as the commercial magnet, suggesting an even higher amount of Fe particles and particles with lower coercivity and susceptibility to overcome this issue.

A soft magnetic material was also used by Bayaniahangar et al. to produce magnetic actuators [62]. They used magnetite nanoparticles with a diameter of 15–20 nm to prepare an ink with polydimethylsiloxane (PDMS) and prepared a hydrogel with Pluronic f-127 as a temporary support for small helical coil structures, which were injected inside the hydrogel bath by a long needle attached to the syringe with the magnetic ink. Afterward, the printed structure was cured at 85 °C, before the hydrogel was liquefied to release the 3D-printed specimens. For optimized ratios of fiber diameter to coil diameter, freestanding coil springs were prepared in this way, which could be used for linear magnetic actuation and as an untethered soft robot.

Magnetite was also used by Ferrara et al. to prepare composites for space-compliant electrical motors [63]. Here, 50 wt% magnetite microparticles were added to a PEEK matrix to prepare filaments for FDM printing. The corresponding magnetic properties were used for a simulation of an axial flux brushless DC electric motor which revealed sufficient torque for small power aerospace applications, combined with up to 50% reduced mass as compared to a common magnet.

Oppositely, some technical applications necessitate quite large saturation magnetization, as it can be found, e.g., in rare-earth-based magnets. Another compound suitable for such applications is Fe_16_N_2_, whose saturation magnetization is similar to those of typical rare-earth permanent magnets, which was embedded in the often-used photoresist SU8 to prepare a 3D-printable ink [64]. Zirhli et al. showed that synthesizing Fe_16_N_2_ flakes by surfactant-assisted high-energy ball milling, reduction and nitridation, and subsequent 3D printing of the Fe_16_N_2_/SU8 ink could be used to prepare a permanent magnet from this highly interesting material.

As this short overview shows, 3D-printed magnets may be useful for several typical technical applications. In addition, 3D-printed magnets are often used in medical applications, as discussed in the next subsection.

### 5.2. Medical Applications

In the medical area, diverse applications are based on magnetic properties of the used materials, or magnetic properties can improve the functionality of the printed objects.

Among the typical medical applications, 3D-printed magnetic scaffolds for tissue engineering are often described in the literature. Zhang et al. combined magnetite nanoparticles with mesoporous bioactive glass and polycaprolactone (PCL) and prepared scaffolds for bone regeneration by 3D printing [65]. For this composite, they found a high compressive strength combined with a high porosity of 60% with uniform pores with a diameter of 400 µm and showed that the specimens could be heated by magnetic hyperthermia, showed good cell proliferation and mineralization of human-bone-marrow-derived mesenchymal stem cells, as well as enhanced osteogenic activity, making them well suitable for bone tissue engineering.

**Figure 8:**
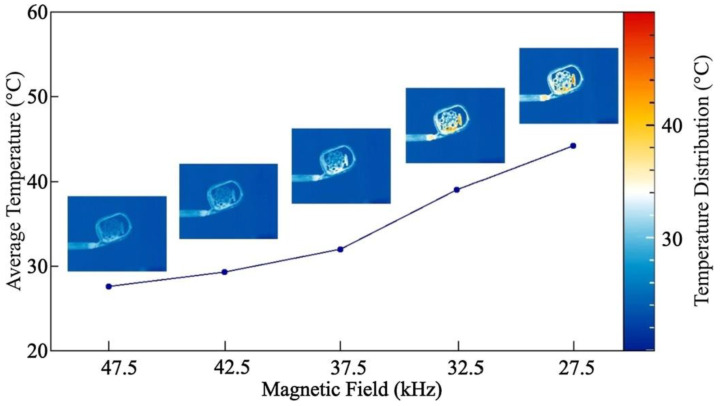
Correlation between average temperature of a 3D-printed specimen and external magnetic field frequency. Reprinted from [66], copyright (2019), with permission from Elsevier.

The heating aspect was also mentioned by Zhang et al. who prepared PLA/magnetite composite filaments for FDM printing [66]. The 3D-printed samples could be heated by applying magnetic alternating fields of different frequencies, as visible in Figure 8. The authors used this effect to trigger the shape memory properties of PLA. With 10% (20%) of magnetite in the composite, they reached recovery times of 14 s (8 s) at the maximum tested magnetic field frequency of 27.5 kHz and suggested using such a composite material especially for bone repair as it could be introduced into bone defects and afterward magnetically heated to completely fill the defect volume.

**Figure 9:**
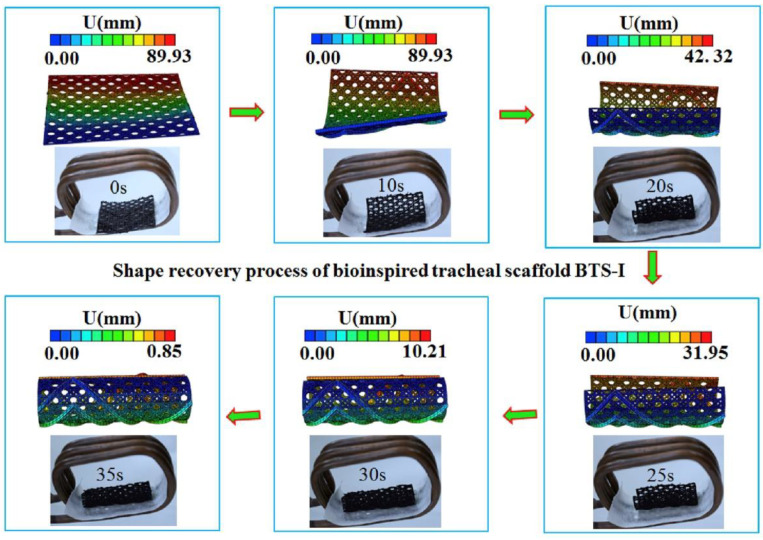
Shape recovery of a bioinspired tracheal scaffold. Reprinted from [67], copyright (2019), with permission from Elsevier.

Magnetic shape memory polymer composites were also prepared by Zhao et al. who prepared tracheal scaffolds inspired by glass sponge skeletons [67]. They also used a PLA/magnetite composite filament, here with a ratio of up to 15 wt% magnetite. In mechanical tests, they found a slight increase in the mechanical strength and the elastic modulus with the addition of magnetite nanoparticles. Shape recovery of the structures was possible within 35 s, as depicted in Figure 9 for simulation and experiment [67].

In addition to scaffolds, there are many more technical applications of 3D-printed magnetic composites in the medical area. Liu et al., e.g., prepared artificial cilia by 3D printing Fe-doped PDMS, which they suggested to be used as a mixer in a lab-on-chip or for legs of endoscopic capsule robots used in the gastrointestinal tract [68]. Von Petersdorff-Campen et al. prepared the magnetic bearing and the magnetic drive coupling of a rotary blood pump by FDM printing [69]. They used a Prusa i3 MK2 with a multi-material upgrade to print self-prepared filaments with 66 wt% NdFeB powder in polyamide 12 with additional polyoxymethylene, dispersing agent, and fumed silica. In this way, they managed to prepare a pump prototype with a maximum rotational speed of 1000 rpm and a flow rate of 3 L/min against a pressure of 6 mmHg. The authors suggested such 3D-printed magnets as a possibility to overcome the limitations of other production methods and to enable more test-driven development in early prototyping stages.

In addition to technical and medical applications, many more possible areas of application for magnetic 3D-printed objects are described in the literature. Some of them are discussed in the next subsection.

### 5.3. Other Applications

In addition to motor parts and similar rigid objects, several applications concentrate on actuators [70,71], magnetic energy harvesters [72], and other rigid parts. Another large area of application is using the magnetic properties of embedded nanoparticles combined with the dielectric properties of the polymeric matrix, e.g., for electromagnetic shielding [73,74] or a tunable ring resonator for the GHz frequency range [75].

**Figure 10:**
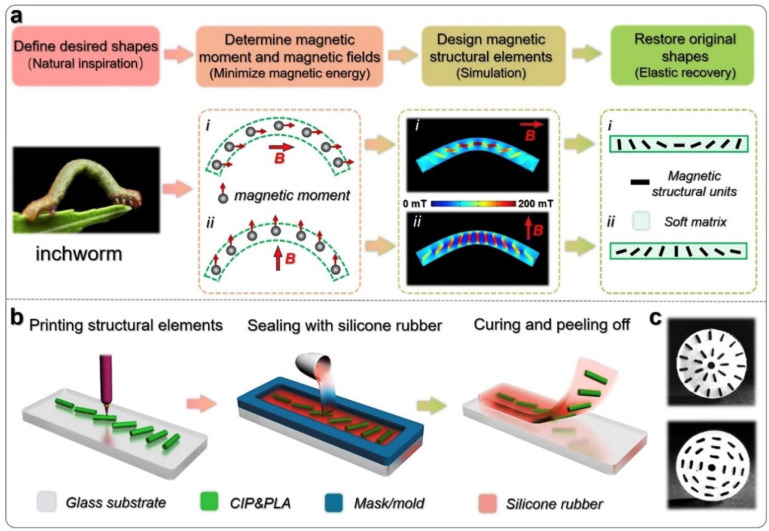
(**a**) Shape-programming strategy for imitation of an inchworm. The deformations are simulated by COMSOL, and the direction of the magnetic field is indicated by the red arrows. (**b**) Three-dimensional printing of magnetic elements and encapsulation by silicone rubber. (**c**) Photos of samples with oriented magnetic structural elements. The radius of the disk samples is 10 mm. Reprinted from [76], copyright (2020), with permission from Elsevier.

In addition to these areas of research and application, soft magneto-active materials are investigated for their possible use, e.g., as grippers or in soft robotics in general. Qi et al. developed a PLA/carbonyl iron particle filament, supported by a silane coupling agent, containing a 1/7 volume ratio of magnetic particles [76]. The FDM-printed shapes were afterward sealed with a silicone rubber, as shown in Figure 10, enabling producing specimens with well-defined magnetic areas in a nonmagnetic matrix. In this way, the authors could bend and relax the inchworm-like structures by applying or switching off an external magnetic field. Other structures with different orientations of inserted magnetic 3D-printed units were also moved by varying external magnetic fields, as shown in different videos in [76].

In a similar way, Zhu et al. used Fe/PDMS composite ink to print 3D structures that could be moved by external magnetic fields [77]. As an example, they prepared a 3D-printed butterfly with fast flapping wings in an alternating external magnetic field. Another inchworm-like soft robot was completely 3D-printed by Joyee and Pan, using a magnetic-field-assisted projection SLA technique (cf., Section 7) [78]. Lee et al. instead used 3D printing to prepare a non-magnetic mold that was afterward filled with magnetic/PDMS fluid, in this way creating different tensegrity structures, e.g., a five-legged tensegrity robot, similar to a starfish, with magnetically actuated movements [79].

Generally, a large research area concentrates on 3D printing magneto-rheological fluids and elastomers. Similar to the aforementioned inchworms with 3D-printed defined magnetic areas [76], 3D printing by a bioprinter or a similar multi-material printer using syringes with needles can be used to prepare magneto-rheological 3D objects. Bastola et al. printed a magneto-rheological fluid and an elastomer matrix that was cured by UV light, so the magneto-rheological fluid was sandwiched between elastomer layers [80,81,82]. In this way, diverse magneto-rheological patterns in a soft polymeric matrix could be printed. The authors showed the variation in stiffness and damping capacity with an external magnetic field, applied in different orientations.

The influence of magnetic annealing on the magneto-active properties of FDM-printed magneto-rheological elastomers was investigated by Fischer et al. [83]. They produced an FDM printing filament by integrating 15 vol% iron powder into Ninjaflex (a thermoplastic elastomer), printed bendable samples, and investigated the bending angle under different applied magnetic fields. They found that annealing before measuring in a uniform magnetic field could increase the magneto-active response, while annealing in a nonuniform magnetic field resulted in a reduced magneto-active response.

While the previous sections concentrate on technical applications and developments, the next one introduces some recent ideas in basic research.

## 6. Basic Research of 3D-Printed Magnetic Structures

**Figure 11:**
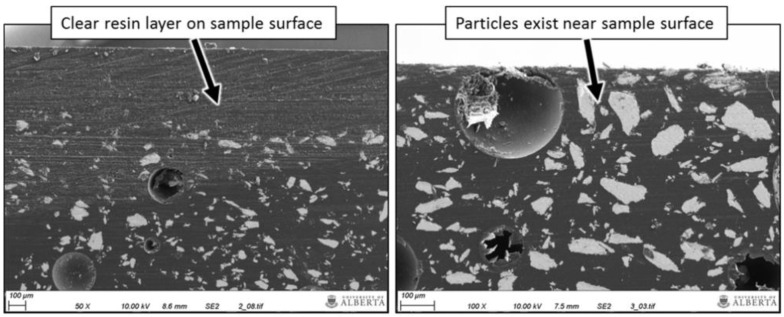
Scanning electron microscopy images of cross-sections of samples with 50 wt% NdFeB and 5% of anti-settling additive (**left**) or 10% of the additive (**right**), respectively. The top of the micrographs corresponds to the samples’ upper surfaces. Reprinted from [86], originally published under a CC-BY license.

As mentioned before, the process of 3D printing can introduce a certain anisotropy in the 3D-printed samples and thus also in their magnetic properties [84]. Bollig et al. investigated the magnetic properties of FDM-printed iron/polymer cubes along different orientations and reported an influence of the outer shell thickness, the fill factor, and the internal layer orientation [85]. Nagarajan et al. investigated the magnetic particle distribution in objects produced by a material-jetting based 3D printing process [86]. For NdFeB powder dispersed in epoxy resin, they suggested using an anti-settling additive and shear-thinning agent in sufficient concentration to avoid undesired settling in the syringe and thus concentration of magnetic particles in the bottom layers of a printed sample, as depicted in Figure 11.

Another often-investigated topic is the combination of magnetic and mechanical properties. Generally, the polymer matrix is mostly responsible for the mechanical integrity of a 3D-printed sample. Thus, while small amounts of fillers may support the stiffness or other mechanical properties, larger amounts of magnetic nano- or microparticles will usually reduce the breaking force and similar parameters [87]. This is why some research groups investigated the mechanical properties of commercially available metal/polymer composites. Laureto et al. used, amongst others, FDM filaments with stainless steel/PLA and magnetic iron/PLA from ProtoPasta and found a strongly increased thermal conductivity, relatively small metal particles shapes such as flakes, and an acceptable air void ratio inside the printed samples [88]. For SLA printing, Joyee et al. prepared a photocurable resin with spherical iron oxide particles of different volume fractions from 4.5–9.5% [89]. They found the influence of the layer thickness to be negligible, while a particle chain orientation parallel to the direction of the applied force was favored.

**Figure 12:**
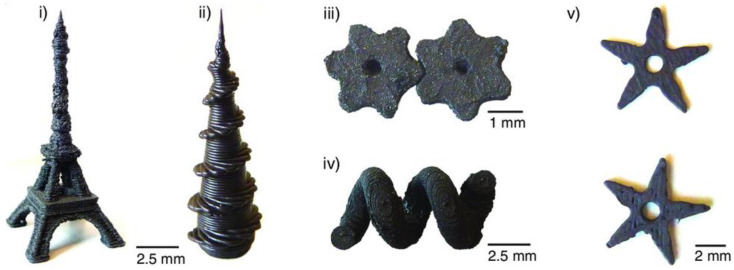
Three-dimensionally printed structures: (**i**) the Eiffel Tower at a scale of 1:1200 printed with NdFeB/PA12, (**ii**) complex helical structure combined with a conical body printed with the SrFe_12_O_19_/PA12, (**iii**) two 3D-printed NdFeB/PA12 gears with magnetization perpendicular to their axis, (**iv**) magnetic helix 3D-printed from NdFeB/PA12 and a water-soluble support material, and (**v**) 3D-printed ninja stars from SrFe_12_O_19_/PA12. Reprinted from [92], originally published under a CC-BY license.

Another typical research question is the resolution that can be reached by different 3D printing processes, a parameter that often needs optimization for printing, e.g., very flat or very small features [90]. Wajahat et al. showed different microstructures, 3D-printed by an extrusion-based technique, from an ink containing 14 wt% magnetite nanoparticles, graphene microflakes, and hydroxypropyl cellulose [91]. For printing, a micronozzle (inner diameter 200 µm) was applied. They investigated applications of these microstructures in 3D-printed electronic devices, e.g., as a magnetic switch, and in a magnet-guided car. A magnetic gear system was printed by Chatzipirpiridis et al. by a common FDM printer from a filament containing NdFeB/PA12 and other commercially available magnetic composite pellets, in addition to different microstructures, as depicted in Figure 12 [92].

Especially, the magnetic properties of such small structures or of composites containing magnetic nano- or microparticles are often simulated [93,94]. Huber et al. prepared an FDM filament from commercially available NdFeB/PA11 powder and printed permanent magnets from it for which they measured and modeled the magnetic flux density in all three spatial directions, finding very good agreement [95]. For much smaller structures, especially open cubes or nano-trees with edge lengths below 1 µm prepared by direct-write fabrication, Keller et al. used a micro-Hall sensor to measure the magnetic stray fields and found very good agreement of these values with micromagnetic and macro-spin simulations [96]. Microscopic eddy currents upon magnetic domain wall movements were simulated and compared with experimental results by Xiang et al. [97].

As these few examples show, experimental and theoretical research of 3D-printed magnetic objects cover a broad range of approaches for diverse applications, with new 3D printing results opening up even more space for new research in this emerging area.

One of the most interesting topics is the anisotropy of the magnetic particles, which can be modified by magnetically supported 3D printing processes, as described in the next section.

## 7. Magnetic-Field-Assisted 3D Printing

Several papers report 3D printing magnetic/polymer or magnetic/resin composites in an external magnetic field to align the magnetic particles or magnetically coated fibers [98,99,100]. Martin et al. used a photopolymer with embedded alumina platelets, coated with electrostatically adsorbed magnetite nanoparticles, for SLA-printing diverse objects inside a structured magnetic field [101]. In this way, they could influence the crack propagation pathways of the loaded specimens by modifying the orientation of the magnetic alumina platelets.

Interestingly, also carbon fibers can be oriented in a strong magnetic field due to their large diamagnetic anisotropy [102]. Pearson et al. used this behavior to print Onyx, a micro-carbon-filled nylon filament, on a common FDM printer, with or without an external magnetic field. They found larger fiber alignment in the experiment than theoretically predicted and suggested further experimental work and simulations to understand this process better and enable reliable technical application.

**Figure 13:**
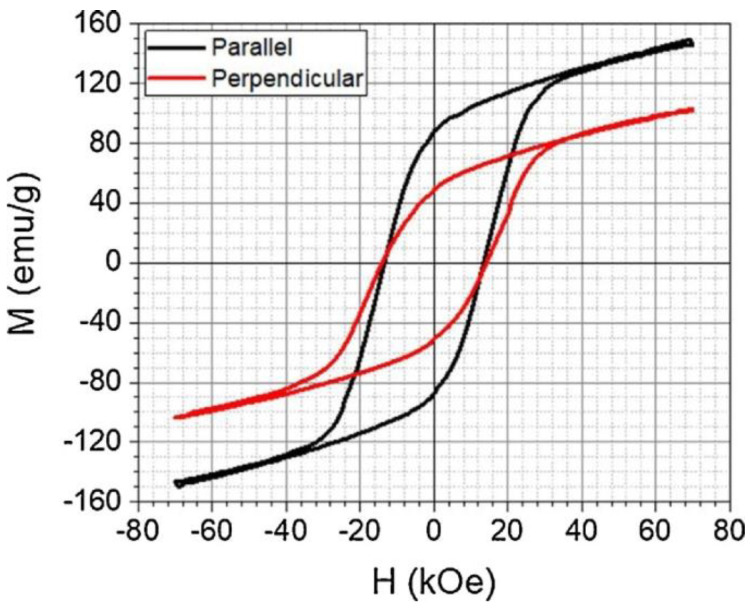
Magnetic characterization of Nd-Fe-B + Sm-Fe-N/Nylon12 65 vol% sample aligned at 230 °C nozzle temperature and 1.5 A alignment current. Reprinted from [103], copyright (2020), with permission from Elsevier.

Using NdFeB and SmFeN powders bonded in PA12 FDM filament, Sarkar et al. found a clear difference in the magnetic hysteresis loops of the 3D-printed samples, measured parallel and perpendicular to the magnetic field direction applied during printing, as depicted in Figure 13 [103].

Generally, the viscosity of a resin is much lower than the viscosity of an FDM polymer in its molten state, making resin-based 3D printing techniques more attractive for magnetic-field-assisted 3D printing [104]. Many studies, thus, describe magnetic-field-assisted 3D printing of photocurable resins, e.g., containing steel fibers [105,106], NdFeB powder [107], nickel-coated carbon fibers [108], carbonyl iron particles [109], or strontium ferrite powder [110]. In this way, not only can the mechanical properties of the final 3D printed objects be modified, but their magnetic anisotropy also allows for using them for special applications in sensing and actuation or even autonomous systems [111,112].

## 8. Conclusions

Three-dimensional printing techniques offer new possibilities to create magnetic objects on different length scales, with hard or soft magnetic materials, aligned or isotropic magnetic properties. The possibility to prepare new shapes without long preparation times enables new applications of such 3D-printed magnets. On the other hand, the polymer matrix clearly limits the possible magnetic properties as saturation magnetization is reduced and coercive fields are varied, depending on the amount of magnetic material in the composite and the sizes and shapes of the embedded magnetic particles; mechanical properties differ significantly from those of a common sintered, purely metallic magnet. Both these limitations have to be taken into account when 3D-printed magnetic composites are developed for specific applications.

This review gives a brief overview of this emerging field of research and development. It can be used to identify the recent trends of research and development, such as 4D printing using magnetic particles for induction heating of shape memory polymers, magnetic scaffolds for tissue engineering, multi-functional 3D-printed objects in which magnetic properties are combined with improved mechanical or other properties, and finally magnetic-field-assisted 3D printing on the technological side.

We hope to inspire more researchers to start working on 3D-printed magnets in experiment and simulation. From the technological side, especially, fiber/matrix adhesion is not often mentioned, so further research on this topic may strongly improve the magnetic/polymer composites’ mechanical properties. From the application side, cancer treatment by hyperthermia therapy, using 3D-printed scaffolds containing magnetite nanoparticles in an oscillating magnetic field, should be investigated further to save lives. Finally, basis research is necessary to enable new findings in addition to the ongoing technical developments, which may result in completely new insights and subsequent applications.

## Data Availability

Not applicable.
